# Decisive role of P42/44 mitogen-activated protein kinase in Δ^9^-tetrahydrocannabinol-induced migration of human mesenchymal stem cells

**DOI:** 10.18632/oncotarget.22517

**Published:** 2017-11-20

**Authors:** Ellen Lüder, Robert Ramer, Kirsten Peters, Burkhard Hinz

**Affiliations:** ^1^ Institute of Pharmacology and Toxicology, Rostock University Medical Center, Rostock, Germany; ^2^ Department of Cell Biology, Rostock University Medical Center, Rostock, Germany

**Keywords:** migration, stem cells, THC, p42/44 MAPK

## Abstract

In past years, medical interest in Δ^9^-tetrahydrocannabinol (THC), the major psychoactive ingredient of the Cannabis plant, has been renewed due to the elucidation of the endocannabinoid system and diverse other receptor targets involved in biological cannabinoid effects. The present study therefore investigates the impact of THC on the migration of mesenchymal stem cells (MSCs) which are known to be involved in various regenerative processes such as bone healing. Using Boyden chamber assays, THC was found to increase the migration of adipose-derived MSCs. Migration by THC was almost completely suppressed by the CB_1_ receptor antagonist AM-251 and to a lesser extent by the CB_2_ receptor antagonist AM-630. By contrast, the TRPV1 antagonist capsazepine as well as the G protein-coupled receptor 55 (GRP55) agonist O-1602 did not significantly interfere with the promigratory effect of THC. Furthermore, increased migration by THC was fully suppressed by PD98059, an inhibitor of p42/44 mitogen-activated protein kinase (MAPK) activation, and was accompanied by a time-dependent activation of this pathway accordingly. In line with the migration data, additional inhibitor experiments pointed towards a decisive role of the CB_1_ receptor in conferring THC-induced activation of p42/44 MAPK. Collectively, this study demonstrates THC to exert a promigratory effect on MSCs via a CB_1_ receptor-dependent activation of p42/44 MAPK phosphorylation. This pathway may be involved in regenerative effects of THC and could be a target of pharmacological intervention.

## INTRODUCTION

Since the discovery of the endocannabinoid system encompassing cannabinoid-activated receptors as well as their endogenously synthesized agonists, an increasing pharmacological interest has been raised in these targets. Of the numerous studies published in this field, the cannabinoid receptors CB_1_ and CB_2_, the transient receptor potential vanilloid 1 (TRPV1) as well as an orphan receptor, G protein-coupled receptor 55 (GPR55), have been demonstrated to be involved in regenerative effects of cannabinoids [1–4]. Particularly, the promigratory impact of cannabinoids on diverse cell types has been identified as part of their tissue protective and regenerative effects such as vasculoprotection [5] as well as corneal [6] and colonic wound healing [7]. Moreover, recent research on cannabinoids´ regenerative effects has been set on bone healing properties associated with cannabinoid receptors and GPR55 signaling [3, 8–10].

As probable cellular targets of regenerative pharmacotherapeutical approaches, mesenchymal stem cells (MSCs) have gained considerable interest due to their multilineage properties [11–13] and thus their ability to support healing processes of the injured myocardium [14], injured spots of the eye [15] as well as disrupted bone tissue [16–18]. Recently, the non-psychoactive cannabinoid cannabidiol (CBD) was demonstrated to confer enhanced migration and osteogenic differentiation of MSCs [19]. In the latter study, CBD-induced migration was mediated via antagonistic action at the GPR55 and agonistic action at the CB_2_ receptor [19]. Related thereto, the mitogen-activated protein kinase (MAPK) p42/44, acting as intracellular key signaling enzyme that modulates stem cell migration [20, 21], has been demonstrated to confer increased stem cell migration by CBD and inhibitors of endocannabinoid degradation [19, 22].

In view of the missing knowledge in this field it is likewise of certain interest to evaluate beneficial or adverse impacts of the major psychoactive component of Cannabis, Δ^9^-tetrahydrocannabinol (THC), on regenerative cells of the human body. Such investigations are warranted to emphasize potential novel indications for cannabinoids but also in view of the already existing therapeutic use of THC-containing drug formulations (for review see [23]). Accordingly, dronabinol (INN for THC) is approved by the US Food and Drug Administration (FDA) for the indications chemotherapy-induced nausea and vomiting (CINV) as well as for anorexia associated with weight loss in patients with acquired immunodeficiency syndrome [23]. Furthermore, nabilone, a synthetic THC analogue, is approved by the FDA to treat CINV [23]. Nabiximols, a standardized Cannabis extract containing approximately equal quantities of THC and CBD, is approved for treatment of multiple sclerosis-associated spasticity by the European Medicines Agency (EMA) as well as for treatment of neuropathic multiple sclerosis-associated and opioid-resistant cancer-related pain by Health Canada [23]. In addition, extracts from Cannabis sativa L. containing THC and CBD have received orphan drug designation for treatment of glioma by both FDA and EMA.

To get further insights into the regenerative action of cannabinoids and to provide evidence for a hitherto unknown potential pharmacological effect of THC, the present study addressed the impact of this cannabinoids on stem cell migration and the functional contribution of cannabinoid-activated receptors as well as p42/44 MAPK to this process. Here, we report THC to confer enhanced migration of MSCs via p42/44 MAPK that becomes predominantly activated by the CB_1_ receptor.

## RESULTS

### Promigratory effect of THC on human MSCs

**Figure 1:**
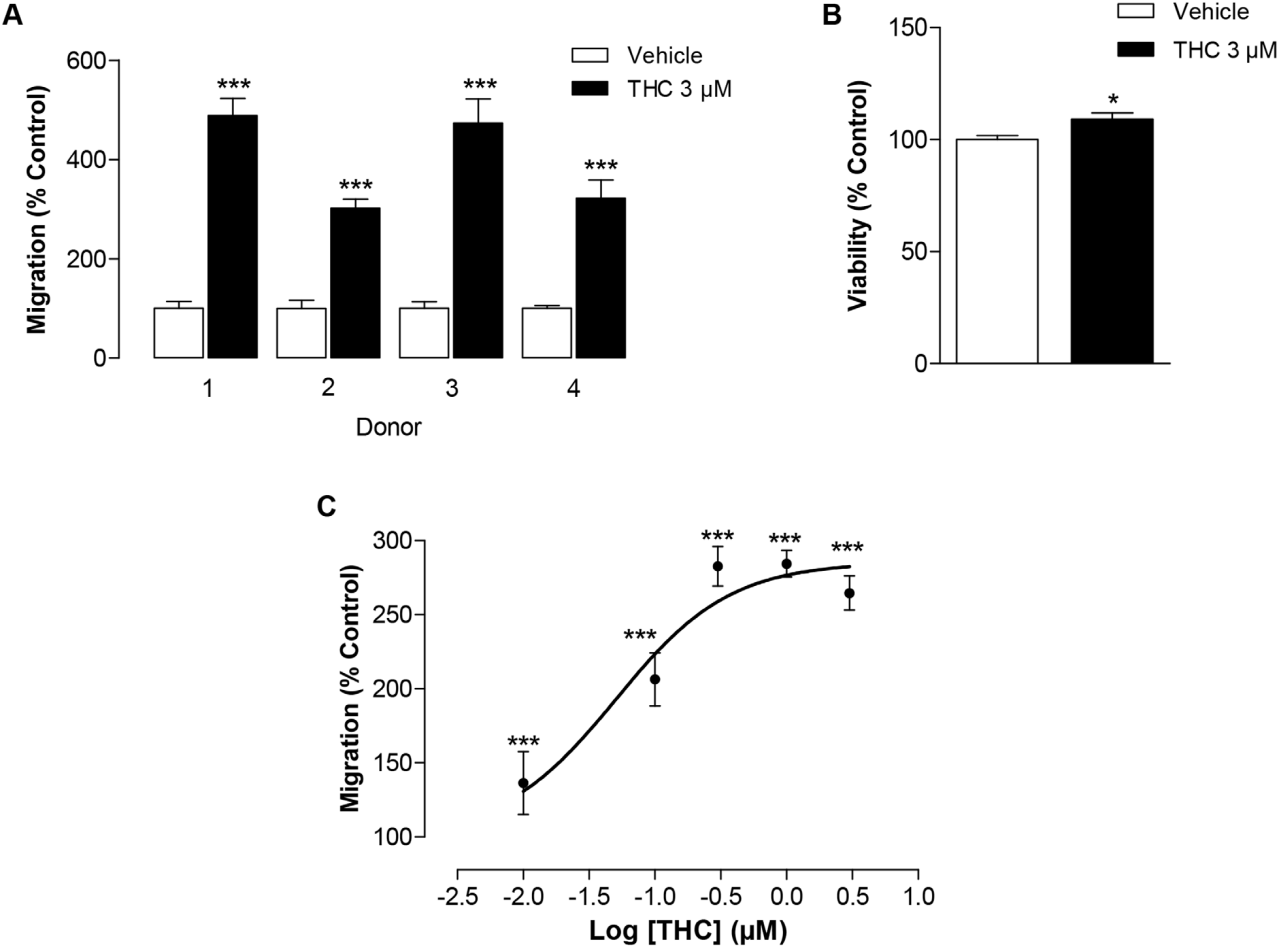
Impact of THC on the migration of MSCs **(A)** Interdonor variability of THC's effect on migration in Boyden chamber assays. MSCs from 4 different donors were incubated with 3 μM THC or vehicle for 6 h. **(B)** Modulation of cellular viability by THC. MSCs were incubated with 3 μM THC or vehicle for 6 h and the viability was analyzed by WST-1 test. **(C)** Concentration-dependent effect of THC on the migration of MSCs in the Boyden chamber assay. Data are expressed as percentage of vehicle-treated controls (set as 100%) and represent means ± SEM of n = 4 (A, C) or n = 4-8 (B) incubations with cells from 1 donor. ^*^P < 0.05, ^***^P < 0.001 vs. corresponding vehicle control, Student's t test.

Initial Boyden chamber assays were performed to monitor interdonor variability. According to Figure 1A, the migration of MSCs obtained from four different donors was shown to be increased by a 6-h incubation with 3 μM THC with a resulting 3- to 4.9-fold stimulation as compared to vehicle-treated cells. To rule out the possibility that enhanced migration of MSCs in response to THC was an unspecific effect due to increased cellular viability, additional WST-1 tests were performed in 96-well plates. As shown in Figure 1B, THC only slightly increased viability in a manner not comparable to its pronounced promigratory effect. The enhanced migration of MSCs by THC appeared as concentration-dependent effect reaching saturation up from 0.3 μM (Figure 1C). The half maximal effective concentration (EC_50_) of this effect was 0.05 μM with a calculated top plateau at 285.5%.

### Role of cannabinoid-modulated receptors in the promigratory effect of THC

To determine the upstream targets of THC-induced migration, MSCs were treated with THC in the presence of antagonists to the CB_1_ receptor (AM-251), CB_2_ receptor (AM-630) and TRPV1 (capsazepine). Recent findings from our group revealed inhibition of GPR55 to enhance MSC migration and the GPR55 receptor agonist 0-1602 to abrogate the promigratory impact of CBD [19]. For this reason, 0-1602 was likewise tested for a probable inhibitory impact on THC´s promigratory effect. Receptor antagonists and O-1602 were used at concentrations of 1 μM that have been shown to be sufficient to modulate the respective receptor activity [19, 24, 25].

**Figure 2:**
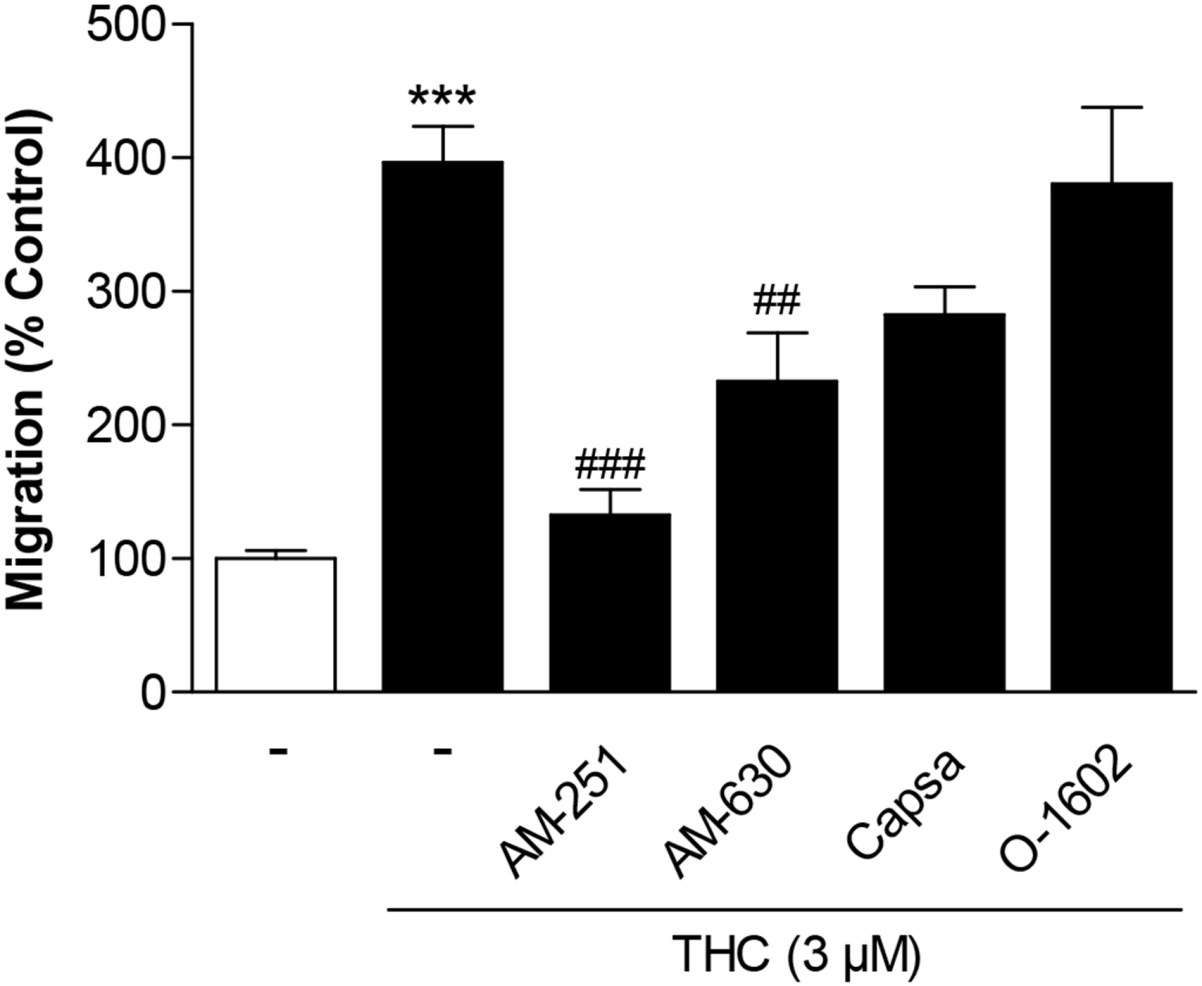
Involvement of cannabinoid-modulated receptors in the promigratory effect of THC on MSCs Cells were pretreated for 1 h with AM-251 (CB_1_ antagonist), AM-630 (CB_2_ antagonist), capsazepine (Capsa, TRPV1 antagonist) or O-1602 (agonist at GPR55) at 1-μM concentrations prior to stimulation of cells with vehicle or 3 μM THC. Migration was analyzed after a 6-h stimulation period in Boyden chamber assays. Data are expressed as percentage of vehicle-treated controls (set as 100%) and represent means ± SEM of n = 16 incubations with cells from 4 donors (4 incubations per donor). ^***^P < 0.001, vs. vehicle control; ^##^*P* < 0.01, ^###^*P* < 0.001 vs. THC-treated cells, one-way ANOVA plus post hoc Bonferroni test.

However, according to the results shown in Figure 2, only AM-251 and AM-630 were found to confer inhibition of THC´s promigratory action with the CB_1_ antagonist exerting the most prominent effect. By contrast, capsazepine as well as O-1602 did not significantly interfere with the promigratory effect of THC (Figure 2). Notably, basal migration of MSCs was recently shown to be left virtually unaltered by the used 1-μM concentrations of AM-251, AM-630 and O-1602 [19], while an equimolar concentration of capsazepine elicited a significant decrease of basal MSC migration [19, 22].

### Role of p42/44 MAPK in the promigratory effect of THC

**Figure 3:**
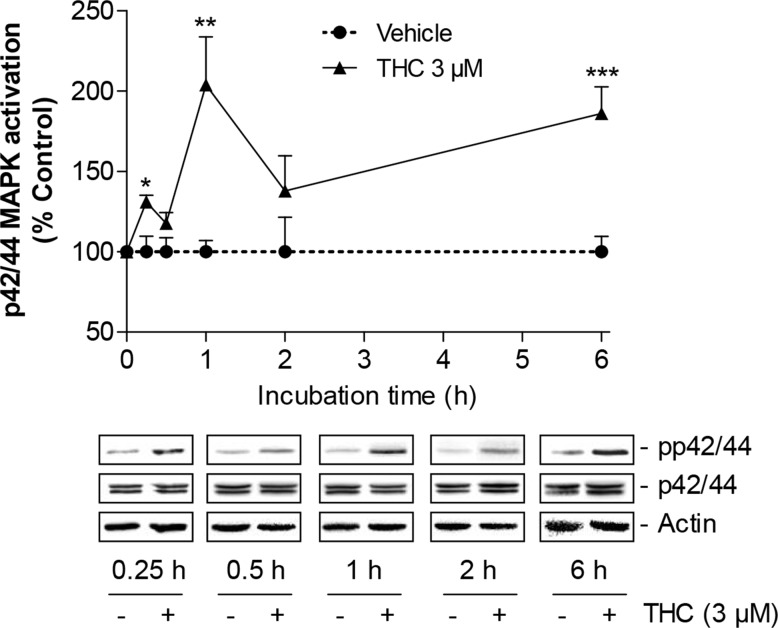
Time-course of p42/44 MAPK activation by THC in MSCs Western blot analysis of p42/44 MAPK activation in MSCs treated with 3 μM THC or vehicle over a 6-h incubation period. The time-course graph above the representative blots indicates densitometric analysis of phospho-p42/44 normalized to p42/44 MAPK. Data are expressed as percentage of vehicle-treated controls (set as 100%) and represent means ± SEM of n = 3 incubations with cells from 1 donor with the exception of the 1- and 2-h values where data were calculated from n = 6 incubations with cells from 2 donors (3 incubations per donor). ^*^P < 0.05, ^**^P < 0.01, ^***^P < 0.001 vs. corresponding vehicle control, Student's t test.

The involvement of p42/44 MAPK in THC-induced migration was investigated contributing to recent findings that indicated this kinase as a major intracellular key regulator of stem cell migration [19–22]. In a first experiment, THC was shown to cause a time-dependent activation of p42/44 MAPK (Figure 3) with a maximum peak following a 1-h incubation period and a delayed activation of p42/44 MAPK after 6 h.

**Figure 4:**
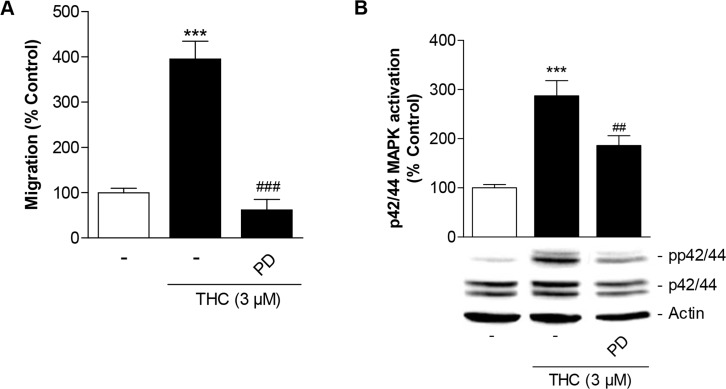
Involvement of p42/44 MAPK activation in THC-induced migration of MSCs Cells were pretreated with the inhibitor of p42/44 MAPK activation, PD98059 (PD, 1 μM), for 1 h and subsequently treated with 3 μM THC or vehicle for another 6 h **(A)** or 1 h **(B)** before quantification of migration in Boyden chamber assays (A) or analyses of p42/44 MAPK activation by Western blot analysis (B). The histogram above the representative blots in (B) indicates densitometric analysis of phospho-p42/44 normalized to p42/44 MAPK. Data are expressed as percentage of vehicle-treated controls (set as 100%). Data in (A) represent means ± SEM of n = 8 incubations with cells from 2 donors (4 incubations per donor). Data in (B) are means ± SEM of n = 14 incubations with cells from 4 donors (3 to 4 incubations per donor). ^***^P < 0.001 vs. corresponding vehicle control, ^##^*P* < 0.01, ^###^*P* < 0.001 vs. THC-treated cells, one-way ANOVA plus post hoc Bonferroni test.

Next, Boyden chamber experiments were carried out to assess the impact of the inhibitor of p42/44 MAPK activation, PD98059, on THC-induced migration of MSCs. The increased migration by THC was fully suppressed by PD98059 (Figure 4A). In line with this data, the efficacy of PD98059 as inhibitor of p42/44 MAPK activation was confirmed by proving its inhibitory action on THC-induced p42/44 MAPK phosphorylation (Figure 4B).

### Role of cannabinoid-modulated receptors in THC-induced p42/44 MAPK activation

**Figure 5:**
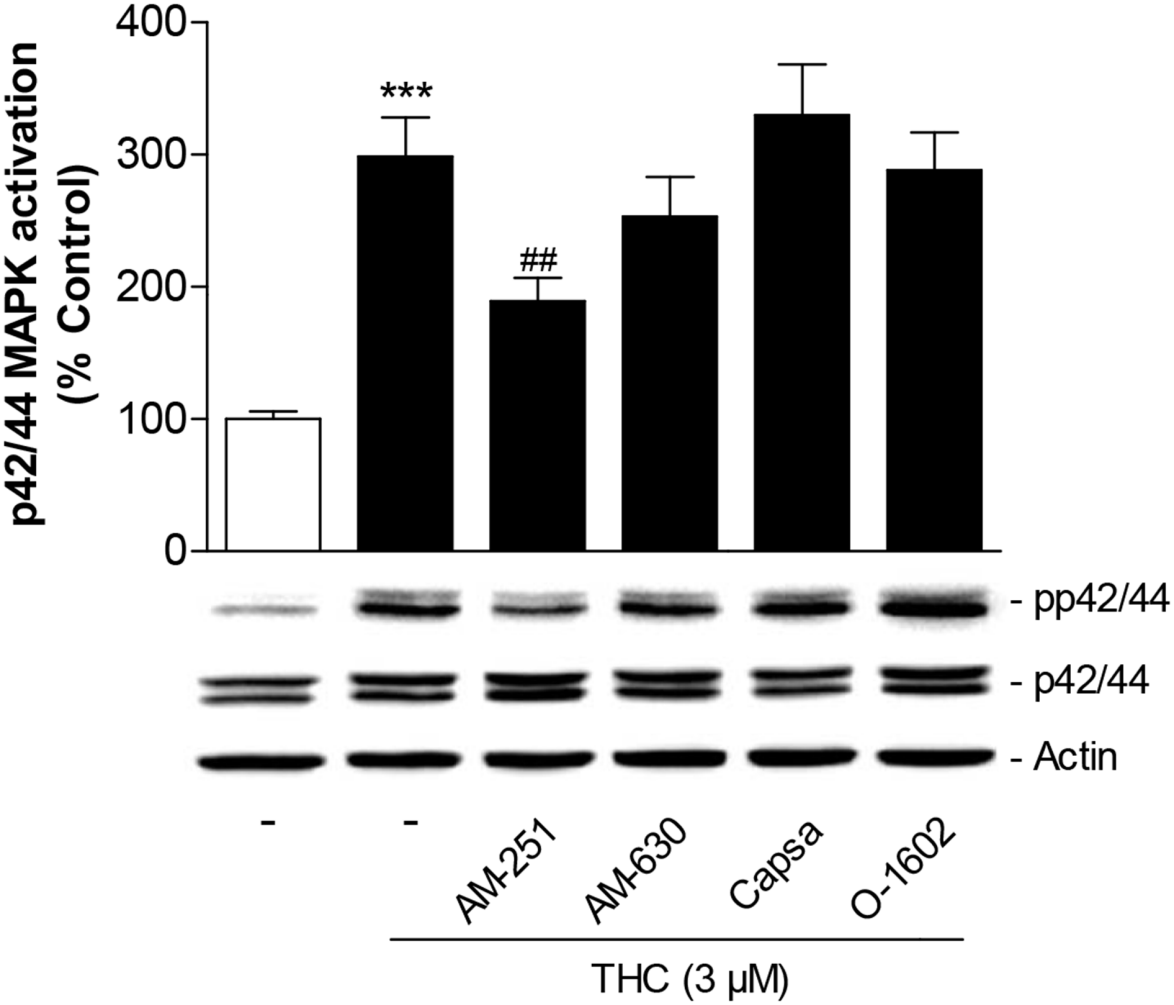
Role of cannabinoid-modulated receptors as upstream targets of p42/44 MAPK activation by THC in MSCs Cells were pretreated for 1 h with AM-251 (CB_1_ antagonist), AM-630 (CB_2_ antagonist), capsazepine (Capsa, TRPV1 antagonist) or O-1602 (agonist at GPR55) at 1-μM concentrations prior to addition of vehicle or 3 μM THC for another 1 h. Activation of p42/44 MAPK was analyzed by Western blot analyses. The histogram above the representative blots indicates densitometric analysis of phospho-p42/44 normalized to p42/44 MAPK. Data are expressed as percentage of vehicle-treated controls (set as 100%) and represent means ± SEM of n = 17 incubations with cells from 5 donors (3 to 4 incubations per donor). ^***^P < 0.001, vs. vehicle control; ^##^*P* < 0.01 vs. cells treated with THC, one-way ANOVA plus post hoc Bonferroni test.

To investigate the involvement of cannabinoid-modulated receptors in the THC-mediated increase of p42/44 MAPK phosphorylation, cells were again pretreated with antagonists to CB_1_, CB_2_, TRPV1 as well as with an agonist at the GPR55. In line with the migration data, the CB_1_ antagonist AM-251 was shown to significantly interfere with p42/44 MAPK phosphorylation by THC (Figure 5). A measurable albeit statistically not significant inhibition was likewise observed with the CB_2_ antagonist AM-630 (Figure 5). On the other hand, both the TRPV1 antagonist capsazepine as well as the GPR55 agonist O-1602 left THC-induced activation of p42/44 MAPK virtually unaltered (Figure 5). The antagonists and O-1602 did not significantly interfere with the basal activation of p42/44 MAPK in MSCs when used at final concentrations of 1 μM as previously described [19].

## DISCUSSION

Our findings demonstrating a promigratory impact of THC are in line with several recent studies indicating cannabinoid compounds to support the migration of regenerative cell types to sites of tissue damage [5–7, 19, 22]. Although MSCs were described to respond heterogeneously to different inputs such as pharmacological challenges [12, 26, 27], initial experiments revealed a relatively consistent promigratory impact of THC that approximately ranged between a 3- to 4.9-fold stimulation as compared to vehicle-treated cells from the respective donor. Thus, these analyses emerged the enhancement of migration as a rather stable and reproducible response of MSCs to THC.

The promigratory action of THC did not appear as unspecific result of enhanced viability as indicated by the corresponding WST-1 tests. Accordingly, the THC-modulated viability, although being significantly enhanced, appeared vanishing as compared to the profound promigratory impact.

Concerning effects of cannabinoid compounds on stem cell migration, we have recently provided evidence for the cannabinoid CBD and for inhibitors of the endocannabinoid-degrading enzyme fatty acid amide hydrolase (FAAH) to induce migration of MSCs in a p42/44 MAPK-dependent manner [19, 22]. In case of CBD, the promigratory impact was mediated predominantly by activation of the CB_2_ receptor and inhibition of GPR55 [19]. In case of FAAH inhibition this effect was found to be causally linked to activation of PPARα [22]. For THC, we here provide data suggesting a likewise p42/44 MAPK-dependent promigratory mechanism of action on MSCs that, however, required a different activation pattern of the respective receptors. Thus, THC preferentially conferred induction of p42/44 MAPK as well as p42/44-MAPK-dependent migration via CB_1_ receptor activation. There are several lines of evidence supporting this notion. First, an inhibitor of p42/44 MAPK activation, PD98059, was found to suppress the THC-induced migration of MSCs, suggesting a contribution of this pathway to the observed promigratory effect of THC. Notably, PD98059 was used at a concentration that was sufficient to profoundly suppress the THC-induced p42/44 MAPK activation in our experimental setting. Second, an antagonist to the CB_1_ receptor, AM-251, caused a clear inhibition of both THC-induced p42/44 MAPK activation and migration. This data strongly support the notion that the CB_1_ receptor represents the predominant upstream target of THC-induced p42/44 MAPK activation and subsequently enhanced migration. Third, the CB_2_ receptor antagonist AM-630 caused partial inhibitions of both THC-induced migration and p42/44 MAPK activation with both effects appearing to a lesser extent that than the respective inhibitory impact of AM-251. Fourth, an agonist at the GPR55, O-1602, as well as the TRPV1 antagonist capsazepine left the promigratory and p42/44 MAPK-activating properties of THC virtually unaltered. Taken together, the antagonists of cannabinoid-activated receptors and O-1602 were shown to elicit comparable patterns of THC-induced migration and p42/44 MAPK activation.

The data presented here are in agreement with our recent observations indicating cannabinoid compounds to exert promigratory effects on MSCs via p42/44 MAPK [19, 22]. Noteworthy, the mode of p42/44 MAPK activation by THC was found to be biphasic which is in line with cannabinoid-induced p42/44 MAPK activation in neuroglioma cells [28]. In the latter study, the endocannabinoid analogue R(+)-methanandamide was shown to confer an early peak after 15 min and a delayed peak after 4 and 8 h. Similar biphasic p42/44 MAPK activations were reported for doxorubicin in neuronal cells [29], for catechol metabolites of 17β-estradiol in pregnancy-derived ovine uterine artery endothelial cells [30] and uridine 5´-triphosphate-treated Schwannoma cells [31]. In the latter investigation biphasic p42/44 MAPK regulation was likewise associated with increased cellular migration [31].

Although THC has been shown to act as a partial agonist at both CB_1_ and CB_2_ [32], our data revealed CB_1_ as the pivotal receptor involved in inhibition of MSC migration and activation of p42/44 MAPK by this cannabinoid. Noteworthy, biological responses of THC may be influenced by the density and coupling efficiencies of these receptors [32]. Furthermore, in a previous study THC was found to elicit a potent CB_1_-mediated adenylylcyclase inhibition (EC_50_ of 11.0 ± 2.1 nM), while a merely 21% inhibition of CB_2_-coupled adenylylcyclase was detected for THC at 1 μM [33]. Another investigation even reported THC to antagonize agonist-induced inhibition of adenylyl cyclase mediated by CB_2_ [34]. The reason why CBD but not THC elicits a CB_2_-dependent inhibition of MSC migration remains unclear. As a matter of fact, CBD exerts a lower affinity to CB receptors than THC with some studies showing a higher affinity of CBD to CB_2_ as compared to CB_1_ [35, 36]. However, despite acting as an inverse agonist/antagonist at these receptors, an activation of CB_2_ by CBD cannot be excluded at present [32]. Accordingly, CB_2_ antagonists have been shown to inhibit CBD-induced inhibition of chemotaxis of murine macrophages [37] as well as antiproliferative effects of CBD on glioma cells [38]. In addition, CBD may elicit effects via inhibition of the endocannabinoid-degrading FAAH [39] and subsequent endocannabinoid-driven receptor signaling. Interestingly, such indirect receptor activation has recently been likewise shown for the endocannabinoid-like substance palmitoyl serine [40].

In the present investigation the THC-induced migration was not inhibited by the GPR55 agonist O-1602, whereas in a recent study O-1602 significantly suppressed CBD-induced stem cell migration and MAPK activation [19]. Again, this may be due to different receptor affinities. Whereas THC does not elicit an antagonistic action at the GPR55 [41], CBD appears to act as GPR55 antagonist [3, 19, 41, 42]. Finally, the missing effect of the TRPV1 antagonist capsazepine on the THC-induced migration was not surprising due to the low affinity of THC to TRPV1 [43]. Notably, capsazepine was recently reported to elicit an inhibitory action on basal migration of MSCs [19, 22] and to inhibit migration of other cell types such as monocytes [44] and hepatoblastoma cells [45]. In contrast to the basal inhibition of MSC migration by capsazepine, the receptor antagonists AM-251 and AM-630 were recently found to leave migration of MSCs virtually unaltered [19, 22].

Concerning the effects of THC on cellular migration, several studies are in contrast to the findings presented here. Accordingly, THC was found to confer inhibition of migration of murine microglia cells [46], cholangiocarcinoma cells [47], human monocytes [48] and human trabecular meshwork cells [49]. On the other hand, one study reported THC to induce migration of human endometrial cells via activation of GPR18 [50]. In the latter study, O-1602 revealed as highly potent full agonist at GPR18 as measured by p42/44 MAPK activation in stably GPR18-transfected HEK293 cells [50]. However, in a recent study from our group [19], O-1602 did not exert a significant impact on both basal migration of MSCs as well as p42/44 MAPK phosphorylation in these cells. Thus, a contribution of GPR18 to cellular migration seems to depend on cell type and receptor density. Taken together, the impact of THC on cellular migration seems to be a cell type-specific phenomenon that has not been addressed in MSCs before.

Concerning the effect of THC on the viability of MSCs, a recent publication addressed the sensitivity of MSCs obtained from bone marrow of Wistar rats toward THC. In these experiments rat MSCs challenged with 1 μM THC for 2 weeks exhibited reduced metabolic activity and increased apoptosis [51]. With respect to our results demonstrating a 6-h treatment of human MSCs with 3 μM THC to induce migration while exerting no cytotoxic action, this diverging data may be due to the different treatment times and/or the species that MSCs are derived from.

In view of the CB_1_-dependent promigratory action of THC on MSCs shown here, it is tempting to speculate that this hitherto unknown action may probably be involved in regenerative or protective processes such as bone formation. In line with this notion, the CB_1_ receptor was demonstrated to protect against age-related bone loss by regulating adipocyte and osteoblast differentiation of bone marrow stromal cells [52]. However, experimental data are contradictory in this respect. Thus, in another investigation inhibition of the CB_1_ receptor was found to be associated with resistance to ovariectomy-induced bone loss via inhibition osteoclast differentiation [8]. Finally, other studies reported enhanced bone loss in wild-type compared with CB_2_ knockout mice [53] and found CBD to gain fracture healing [54].

In the present study, THC exhibited significant promigratory properties even at a concentration as low as 0.01 μM with a calculated EC_50_ of 0.05 μM. In comparison, peak plasma levels of THC in humans were 0.060 μM after pulmonal administration of THC-containing vapours and 0.863 μM after intravenous administration of 0.053 mg THC per kg body weight in healthy volunteers [55]. Oral intake of 15 mg THC and buccal administration of nabiximols with a THC dose of 16.2 mg by Cannabis smokers yielded THC peak plasma levels of 0.045 μM and 0.049 μM, respectively [56]. Accordingly, the data presented here provide evidence for THC to act on the migration of MSCs at pharmacologically relevant concentrations.

Collectively and to the best of our knowledge, this is the first study reporting a promigratory impact of THC on MSCs, which may be an additional mechanism in the complex network of regenerative action of cannabinoids.

## MATERIALS AND METHODS

### Materials

THC was obtained from Lipomed (Weil am Rhein, Germany). O-1602 and PD98059 were bought from R&D Systems (Wiesbaden-Nordenstadt, Germany). AM-251 and AM-630 were purchased from Biomol GmbH (Hamburg, Germany). Dulbecco's Modified Eagle's medium (DMEM, high glucose, GlutaMAX^TM^), trypsin-EDTA and penicillin-streptomycin were obtained from Life Technologies GmbH (Darmstadt, Germany). Fetal calf serum (FCS) was from PAN Biotech (Aidenbach, Germany). Dimethyl sulfoxide (DMSO), EDTA, glycerol, glycine, H_2_O_2_, HEPES, MgCl_2_, NaCl, sodium dodecyl sulphate and Tris were bought from AppliChem GmbH (Darmstadt, Germany). Aprotinin, capsazepine, HCl, leupeptin, luminol, orthovanadate, para-cumaric acid, phenylmethylsulfonyl fluoride (PMSF) and Triton^®^ X-100 were obtained from Sigma-Aldrich (Taufkirchen, Germany). Milk powder was obtained from Bio-Rad Laboratories GmbH (Munich, Germany). Tween^®^ 20 was purchased by Carl Roth GmbH (Karlsruhe, Germany).

### Cell culture

Subcutaneous adipose samples were acquired by liposuction. The donors had been informed about the establishment of cellular models from their tissue and had given informed consent. MSCs were isolated from adipose tissue as previously described [19, 57]. Briefly, adipose tissue was digested in collagenase NB4 standard grade solution (6 mg/ml, SERVA, Heidelberg, Germany) with gentle agitation for 30 min at 37°C. The mature adipocyte fraction was separated from the stromal cell fraction by centrifugation at room temperature at 400 × g for 10 min. Subsequently, the stromal fraction was filtered through a 100 μm nylon mesh (BD Falcon^TM^ Cell Strainer; BD Biosciences, Heidelberg, Germany) and centrifuged at room temperature at 400 × g for 10 min. Pellets were resuspended in phosphate-buffered saline (PBS) with 10% FCS and centrifuged once more at room temperature at 400 × g for 10 min. Cells were cultured in DMEM with 10% FCS, 100 U/ml penicillin and 100 μg/ml streptomycin. From these primary cultures, MSCs were isolated via their characteristic expression of CD34 surface antigen, using the Dynabeads^®^ CD34 Positive Isolation Kit (Life Technologies GmbH) on the basis of the manufacturer's instructions. Cells prepared and isolated using the abovementioned procedure were recently tested and confirmed concerning the presence of specific MSC markers such as CD29, CD44, CD105, and CD166 as well as for the absence of CD14/CD68 as monocyte/macrophage markers, and CD31 as endothelial marker [58]. Furthermore, the osteogenic and adipogenic differentiation potentials of these MSCs were recently verified [19, 59].

All experiments were conducted with cells from passage 4. MSCs were seeded at a density of 2 × 10^4^ cells per cm^2^ in all experiments except WST-1 tests (2 × 10^4^ cells per well of a 96-well-plate). Incubations were performed in serum-free DMEM.

Test substances were dissolved in ethanol or DMSO and diluted with PBS to yield final concentrations of 0.01% (v/v) ethanol (for THC) or 0.01% (v/v) DMSO (for AM-251, AM-630, capsazepine, O-1602 and PD98059). As vehicle control PBS containing the respective concentration of ethanol or DMSO was used.

### Analysis of cellular viability

For evaluation of cellular viability 2 × 104 cells per well were seeded into a 96-well plate. Following incubation with 3 μM THC or vehicle for 6 h (Figure 1B), viability was tested by the colorimetric WST-1 assay (Roche Applied Science, Mannheim, Germany). This assay is based on the cleavage of the tetrazolium salt WST-1 (4-[3-(4-Iodophenyl)-2-(4-nitro-phenyl)-2H-5-tetrazolio]-1.3-benzene disulfonate) to formazan by mitochondrial dehydrogenase activity.

### Migration assay

The effect of test substances on the transmigration of MSCs was determined using a modified Boyden chamber assay according to the manufacturer's instructions (BD Biosciences) as described previously [19, 22]. In this assay, cellular motility is monitored by migration through pores of a transwell insert (8 μm pore size) towards a chemoattractant. In brief, 1 × 105 cells per insert were seeded onto the upper side of the transwell inserts and treated with test substances or vehicles for the times indicated. DMEM containing 10% FCS was used as chemoattractant in the companion plate. Following incubation at 37°C and 5% CO_2_ atmosphere for 6h, the non-migrated cells on the upper surface of the inserts were removed with a cotton swab. For calculation of migration, the viability of the migrated cells on the lower side of uncoated chambers was determined by the colorimetric WST-1 test.

### Western blot analysis

For analysis of protein levels, human MSCs were seeded into 6-well plates and grown to confluence. Western blot analyses were performed as described previously [19]. Following incubation with test substances or vehicles for the indicated times, cells were lysed in solubilization buffer [50 mM HEPES pH 7.4, 150 mM NaCl, 1 mM EDTA, 1% (v/v) Triton^®^ X-100, 10% (v/v) glycerol, 1 mM PMSF, 1 μg/ml leupeptin, 0.5 mM orthovanadate and 10 μg/ml aprotinin], homogenized by vigorous mixing for 30 min on ice, and centrifuged at 20,000 × g for 5 min. Total protein concentration was measured using the bicinchoninic acid assay (Pierce^®^, Thermo Fisher Scientific, Bonn, Germany). Proteins were separated on a 10% sodium dodecyl sulfate polyacrylamide gel. Following transfer to nitrocellulose (Carl Roth GmbH) and blocking of the membranes with 5% (w/v) milk powder, blots were probed with specific antibodies raised to β-actin (loading control; Sigma-Aldrich) or p42/44 and phospho-p42/44 MAPK (New England BioLabs GmbH, Frankfurt, Germany). Subsequently, membranes were probed with horseradish peroxidase-conjugated Fab-specific anti-mouse (β-actin) or anti-rabbit IgG (New England BioLabs GmbH). The following antibody dilutions were used: 1:5000 (β-actin) and 1:1000 (p42/44, phospho-p42/44, anti-mouse IgG, anti-rabbit IgG). Antibody binding was visualized using chemiluminescence solution containing 1.25 mM luminol, 200 μM para-cumaric acid, 0.09% (v/v) H_2_O_2_ and 0.0072% (v/v) DMSO in 100 mM Tris-HCl (pH 8.5).

Densitometric analysis of band intensities was achieved by optical scanning and quantifying using the Quantity One 1-D Analysis Software (Bio-Rad Laboratories GmbH). Activation of p42/44 MAPK was calculated by normalizing densitometric values of blots exposed to phospho-specific antibodies to densitometric values of the bands obtained from blots exposed to the respective antibodies against the respective non-phosphorylated kinases.

### Statistics

Comparisons between 2 groups were performed by Student's t test. Comparisons among more than 2 groups were carried out with one-way ANOVA plus post hoc Bonferroni test. In experiments addressing the effects of inhibitors or antagonists on THC-induced effects, indications of statistical evaluations are restricted to comparisons of vehicle versus stimulus alone (indicated as asterisks) and comparisons of stimulus alone versus respective stimulus plus inhibitor/antagonist (indicated as hash signs). Nonlinear regression (Figure 1C) was performed according to the following formula: Y = Bottom + (Top-Bottom)/(1+10^(LogEC_50_-X). Bottom value was determined as the vehicle control (100%) and top was calculated as plateau of the maximal induction of migration in response to the concentrations (X). EC_50_ represents increase of migration halfway between vehicle control (100%) and top. All statistical analyses were undertaken using Prism 5.04 (GraphPad Software, San Diego, USA).

## Abbreviations

AM-251: [N-(piperidin-1-yl)-5-(4-iodo-phenyl)-1-(2,4-dichlorophenyl)-4-methyl-1H-pyrazole-3-carboxamide], selective CB_1_ receptor antagonist
AM-630: [(6-iodo-2-methyl-1-[2-(4-morpholinyl)ethyl]-1H-indol-3-yl) (4-methoxyphenyl)methanone], selective CB_2_ receptor antagonist
CB_1_: cannabinoid receptor 1
CB_2_: cannabinoid receptor 2
GPR55: G protein-coupled receptor 55
MAPK: mitogen-activated protein kinase
MSCs: mesenchymal stem cells
O-1602: 5-methyl-4-[(1R,6R)-3-methyl-6-(1-methylethenyl)-2-cyclohexen-1-yl]-1,3-benzenediol, selective GPR55 agonist
PD98059: 2-(2-amino-3-methoxyphenyl)-4H-1-benzopyran-4-one, inhibitor of p42/44 MAPK activation
TRPV1: transient receptor potential vanilloid 1
WST-1: 4-[3-(4-iodophenyl)-2-(4-nitrophenyl)-2H-5-tetrazolio]-1.3-benzene disulfonate
